# Predicting Outcomes of Penetrating Cardiovascular Injuries at a Rural Center by Different Scoring Systems

**DOI:** 10.21470/1678-9741-2019-0403

**Published:** 2020

**Authors:** Ali Ahmet Arikan, Emre Selçuk, Fatih Avni Bayraktar

**Affiliations:** 1 Department of Cardiovascular Surgery, Kocaeli University Medical Faculty, Kocaeli, Turkey.; 2 Department of Cardiovascular Surgery, Muş State Hospital, Muş, Turkey.

**Keywords:** Rural Hospitals, Vascular System Injuries, Injury Severity Score, Penetrating Wounds, Heart Injuries, Area Under Curve

## Abstract

**Objective:**

To compare the anatomical and physiological scoring systems and the outcomes of surgical management of penetrating cardiovascular trauma at a rural center.

**Methods:**

Seventy-seven patients underwent emergency surgery at our center between January/2012 and October/2018 due to penetrating cardiovascular trauma. Injury Severity Score (ISS), Revised Trauma Score (RTS), New Injury Severity Score (NISS), and Trauma and Injury Severity Score (TRISS) were calculated. The validation of these risk scores to predict mortality was assessed by the area under the receiver operating characteristic curve analysis.

**Results:**

All trauma scores were correlated with mortality. As ISS, NISS, and TRISS values increased and RTS values decreased, the mortality rate increased. The area under the curve (AUC) in the receiver operating characteristic curve analysis was 0.943 for TRISS, 0.915 for RTS, 0.890 for ISS, and 0.896 for NISS (*P*<0.001 for each). Logistic regression analysis revealed that scores were correlated with mortality (*P*<0.001 for each). By investigating cardiac injuries alone, only TRISS and RTS results correlated with mortality for cardiac injuries (Mann-Whitney U test, *P*=0.003 and *P*=0.01, respectively). The AUC was only statistically significant for TRISS and RTS (AUC=0.929, *P*<0.05 for both). For vascular injuries, all the scores were significantly correlated with in-hospital mortality (Mann-Whitney U test, *P*<0.001 for each). TRISS had the highest AUC (AUC=0.946, *P*<0.001).

**Conclusion:**

TRISS has the highest predictivity for in-hospital mortality in patients with penetrating cardiovascular trauma.

**Table:** 

Abbreviations, acronyms & symbols	
AIS	= Abbreviated Injury Scale
AUC	= Area under the curve
CI	= Confidence interval
EDT	= Emergency department thoracotomy
GCS	= Glasgow Coma Scale
ISS	= Injury Severity Score
NISS	= New Injury Severity Score
NPV	= Negative predictive value
OR	= Odds ratio
PPV	= Positive predictive value
ROC	= Receiver operating characteristic
RR	= Respiratory rate
RTS	= Revised Trauma Score
SAP	= Systolic arterial pressure
SBP	= Systolic blood pressure
SD	= Standard deviation
TRISS	= Trauma and Injury Severity Score

## INTRODUCTION

Trauma involving the cardiovascular system is a lifethreatening condition where immediate intervention is critical. Penetrating trauma on the cardiovascular system usually occurs as a result of violence involving firearms or sharp materials and can present with a wide range of clinical severities and heterogeneous accompanying organ injuries. According to Turkish Statistical Institute’s 2017 data, 4.5% of all deaths in the country were due to injury and poisoning, and it is the leading cause of death in the population between the ages of 15 and 34 years^[1,2]^. About 70% of the homicides in the country are related to penetrating mechanisms (impact of firearms or sharp force)^[3]^. The therapy of penetrating cardiovascular injuries is a challenge not only due to the complexity of the anatomical neighbors of cardiovascular structures but also because of its interactions with other organs and systemic results of hemorrhage. Results of cardiovascular trauma in large centers have already been published, but less is known about rural centers^[4]^. Different health politics are used to provide sufficient health care in rural regions worldwide^[5-8]^. In Turkey, cardiac and vascular systems surgeries are both managed by the cardiovascular surgeon, and the rural areas are obligatory service zones for clinicians to give availability and accessibility to health care services in all geographic areas. Due to the regulations of the ambulance organization, all cardiovascular trauma patients are referred to the study center, which is the only center providing a cardiovascular surgeon in the region and is serving a rural area with 400,000 inhabitants^[9]^. The facilities of our study hospital were consistent with the definition of a rural level 3 trauma center^[10]^.

Scoring systems are well-established tools in trauma epidemiology, quality assurance, and outcome prediction. The Abbreviated Injury Scale (AIS) is used to classify blunt and penetrating trauma^[11-14]^. To numerically express the severity of a traumatized patient, the Injury Severity Score (ISS), New Injury Severity Score (NISS), Revised Trauma Score (RTS), and Trauma and Injury Severity Score (TRISS) emerged^[15-18]^.

As no specific tool for the prediction of in-hospital mortality for penetrating cardiovascular trauma in rural centers is wellestablished, correlations of the ISS, NISS, RTS, and TRISS with patient outcomes were investigated. We evaluated the results of both cardiac and vascular traumas to validate a single scoring system for cardiovascular trauma to be used by the cardiovascular surgeon. Additionally, a separate analysis was also performed for cardiac and noncardiac injuries (involving only vascular trauma).

## METHODS

The institution’s approval for retrospective research and the approval from the ethical committee were obtained. This single-center retrospective cohort study included all patients with penetrating cardiac and/or vascular trauma between January/2012 and October/2018 at Muş State Hospital. All the patients’ data were available in the hospital’s electronic records. All surgically treated trauma patients > 14 years old and admitted following penetrating injury to a named blood vessel and/or cardiac injury were included in the study.

Seventy-seven patients met the inclusion criteria. Patients’ demographic data, comorbid diseases, vital signs (pulse rate, respiratory rate, and blood pressure), penetrating trauma mechanism, anatomic location of the injury, accompanying organ injuries, operative procedures, and in-hospital mortality were evaluated. ISS, NISS, RTS, and TRISS (regarding predicting death rate) for penetrating trauma were calculated for each trauma victim. The main characteristics of the scoring systems are presented in Table 1. The mentioned trauma scores of the patients who survived and the ones who did not were compared.

**Table 1:** Variables and calculation of ISS, NISS, RTS, and TRISS.

Scores	Variables	Calculation
ISS	Highest AIS grade in each of the three most severely injured ISS body regions	Sum of [(three most weighted injury per region)^2]^
NISS	Highest three AIS grades of injuries, irrespective of ISS body regions	Sum of [(three most weighted injury)^2]^
RTS	Respiratory rate, systolic blood pressure, Glasgow Coma Scale	Sum of [(RR value) ´ 0.2908; (SBP value) ´ 0.7326; (GCS value) ´ 0.9368]
TRISS	RTS, ISS, age, mechanism of injury (blunt or penetrating)	b = -2.5355 + RTS ´ 0.9934 + ISS ´ -0.0651 + (age.points) ´ -1.1360 Predicted death rate = 1/(1 + e^b^)

The Abbreviated Injury Scale (AIS) defines the severity of trauma for each organ system, and injuries are classified in six groups (minor: 1; moderate: 2, serious: 3, severe: 4, critical: 5, and maximal: 6 points). The corresponding points are used for the calculation of Injury Severity Score (ISS) and New Injury Severity Score (NISS). For ISS, the body is divided into six zones (head and neck; face; chest; abdomen and pelvic contents; extremity and pelvic girdle; external injuries). A value between 0 and 4 is defined for specific ranges of respiratory rate (RR), systolic blood pressure (SBP), and Glasgow Coma Scale (GCS) to calculate the Revised Trauma Score (RTS). Coefficients are used to calculate RTS and Trauma and Injury Severity Score (TRISS). Coefficients defined for penetrating injuries are used in calculation of TRISS in our study

### Statistical Analysis

The categorical variables were presented as counts and frequencies, and continuous variables as mean and standard deviation. Chi-square test or Fisher’s exact test was used for comparison between the categorical variables. Student’s *t*-test or Mann-Whitney U test was used to compare the continuous variables. The performance of the risk scores was assessed using receiver operating characteristic (ROC) curves and univariate logistic regression analysis. Sensitivity, specificity, and positive and negative predictive values for mortality prediction for each score were calculated. A *P*-value of 0.05 was considered statistically significant. The IBM SPSS Statistics software (SPSS, Chicago, Illinois, United States of America), version 23.0, was used for all statistical analyses.

## RESULTS

### Baseline Characteristics

Seventy-seven consecutive patients underwent surgical repair due to penetrating cardiovascular trauma during the study period. About 19% (n=15) of all the patients were female. The mean age of the patients was 28.7±10.7 years (range: 15 to 60 years). There was no significant difference between the ages of the survivors and nonsurvivors (28.6±11.3 *vs.* 27.6±10.6, respectively; *P*=0.72). Three patients had schizophrenia, one patient had arterial hypertension, and no other chronic diseases were present. Injured structures and related mortality are shown in Table 2. About 17% (n=13) of the patients had multiple cardiovascular injuries. About 53% (n=41) of the patients had accompanying organ injuries (17 lung injuries, 12 intra-abdominal organ injuries, eight bone fractures, four liver injuries, and four peripheral nerve damages). About 27% (n=21) of the 77 patients died (two patients with stab wounds and 19 patients with shotgun wounds). About 47% (n=36) of the study population had a shotgun wound, 41% (n=32) had a stab wound, and 12% (n=9) had injuries caused by other sharp materials. None of the six patients with cardiac injuries due to shotgun wounds survived. The patients with shotgun wounds had a higher mortality rate than those with stab wounds (*P*<0.001). The hemodynamic statuses at admission of the survivors and nonsurvivors are given in Table 3. About 61% (n=13) of the nonsurvivors were on cardiopulmonary resuscitation on admission. Six patients with thoracic trauma underwent resuscitative emergency department thoracotomy (EDT). Three of six patients could survive the operation, but only one case could be discharged from the hospital (16% survival). About 51% (n=39) of the patients had ISS > 15; a mortality rate of 26% (n=20) was present among this group. Saphenous vein graft (n=28; 17 patients), prosthetic graft interpositions (n=3), and venous patch repair (n=2) were used. The remaining patients were treated with end-to-end anastomosis or primary repair. Five patients needed fasciotomy after vascular repair; no amputations were required. Teflon-pledgeted mattress sutures (n=12) and a pericardial patch (n=1) were used for cardiac injuries. One patient required coronary artery bypass graft procedure at an external center due to the distal left anterior descending artery injury following suture repair of cardiac injury at the study hospital.

**Table 2:** Patients’ injured structures and related mortality.

	Number of cases	Number of additional non- CV injuries	Mortality (n)
			
Heart*	13	4	7
Thoracic aorta**	2	2	2
Innominate vein	1	1	1
Carotid	1	-	1
Pulmonar vessels	2	2	2
Abdominal aorta***	3	3	3
Vena cava ınferior	5	5	3
Iliac artery***	2	2	1
Femoral vein	3	-	1
Femoral artery^†^	4	-	-
Popliteal artery^††^	4	2	-
Popliteal vein	1	-	-
Tibial artery	4	4	-
Subclavian vein	2	2	-
Axillary artery^†††^	2	1	-
Axillary vein	1	1	-
Brachial artery^‡^	9	1	-
Radial artery	6	4	-
Ulnar artery^‡‡^	3	3	-
Cephalic/basilic vein	2	-	-
External jugular vein	2	-	-
Occipital artery	1	1	-
Internal thoracic artery^‡‡‡^	2	1	-
Intercostal artery	2	2	-

Concomitant injuries of cases with multiple cardiovascular (CV) injuries are marked with symbols. The number of patients with accompanying nonvascular injuries (lung, bowel, liver, bone, or nerves) is enlisted. Mortality among injuries is given as numbers.

* one case with left anterior descending artery injury;

** one case with pulmonary hilar vessel injury;

*** one case with ipsilateral iliac vein injury;

† three cases with ipsilateral femoral vein injury;

†† two cases with ipsilateral popliteal vein injury;

††† two cases with ipsilateral axillary vein injury;

‡ one case with brachial vein injury;

‡‡ two cases with ipsilateral radial artery injury;

‡‡‡ one case with pericardial penetration

**Table 3:** Outcomes of patients according to the measured blood pressures on arrival at the hospital.

Systolic blood pressure (mmHg)	Injury location	Patient (n)	Survivor (n)	Nonsurvivor (n)
Immeasurable	Cardiac	4	-	4
	Vascular	12*	3	9
≤ 60	Cardiac	2	1	1
	Vascular	2**	-	2
60-90	Cardiac	6	4	2
	Vascular	17^†^	15	2
≥ 90	Cardiac	1	1	-
	Vascular	33^††^	32	1

Of all patients, 21% (n=16) had immeasurable blood pressure and required cardiopulmonary resuscitation during transport or following arrival to the hospital, all of them had signs of life at the trauma scene; 5% (n=4) had profound hypotension (≤ 60 mmHg systolic arterial pressure [SAP]) at admission; 30% (n=23) were hypotensive (60-90 mmHg SAP); and only 44% (n=34) were hemodynamically stable ( ≥ 90 mmHg SAP)

* seven truncal, five junction vessel injuries;

** two truncal vascular injuries;

† five junctional, eight truncal, four extremity vascular injuries;

†† five truncal, five junctional, 23 extremity vascular injuries

### Validation of Risk Scores

**Fig. 1:**
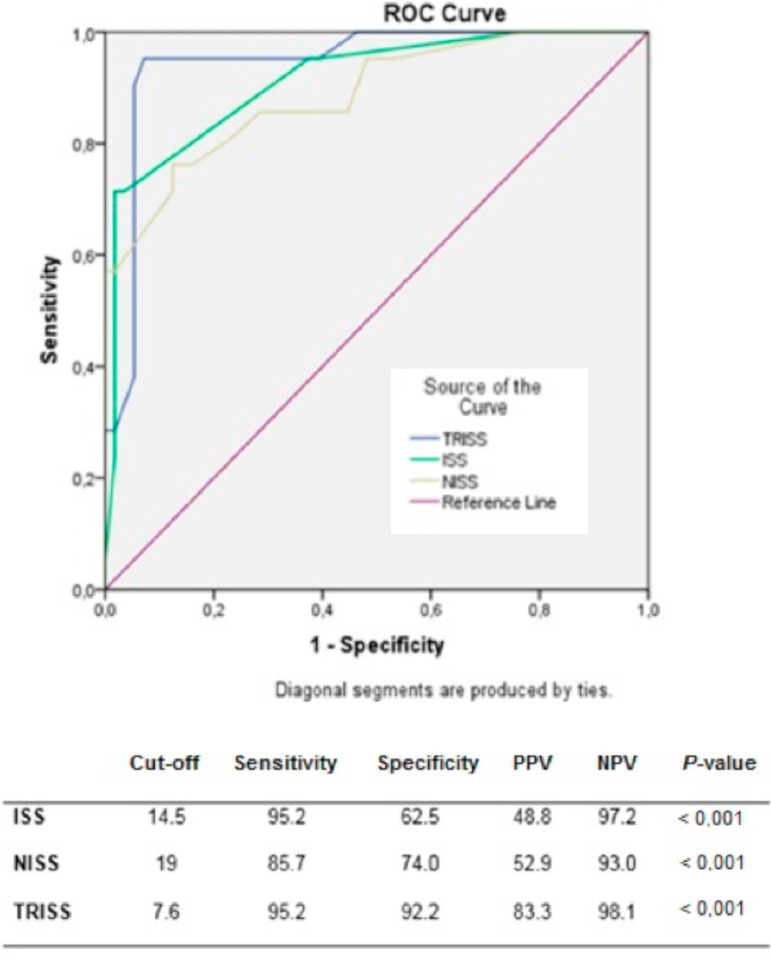
Receiver operating characteristic (ROC) analysis of ISS, NISS, and TRISS for the risk of mortality in cardiovascular injuries. Cut-off values of risk scores in the prediction of mortality. ISS=Injury Severity Score; NISS=New Injury Severity Score; NPV=negative predictive value; PPV=positive predictive value; TRISS=Trauma and Injury Severity Score.

**Fig. 2:**
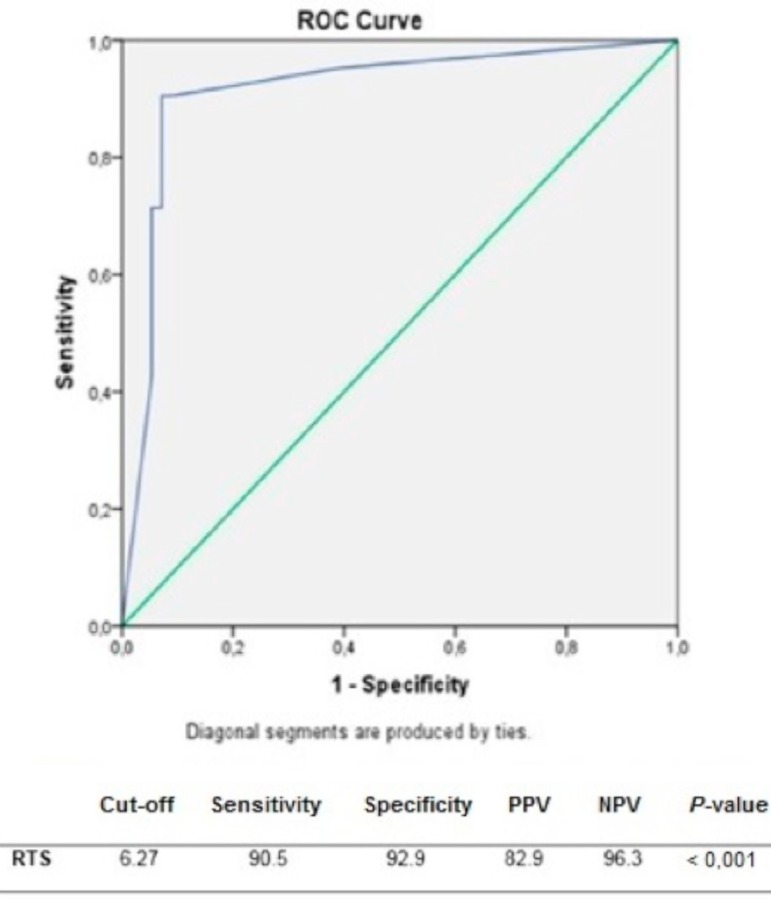
Receiver operating characteristic (ROC) analysis of RTS for the risk of mortality in cardiovascular injuries. Cut-off values of risk scores in the prediction of mortality. NPV=negative predictive value; PPV=positive predictive value; RTS=Revised Trauma Score.

Mean ISS, NISS, TRISS, and RTS of all the patients, survivors and nonsurvivors, are shown in Table 4. The risk scores were compared with the rate of survival. ISS, NISS, TRISS, and RTS were correlated with mortality in our study population (Mann-Whitney U test, *P*<0.001 for each). When ISS, NISS, and TRISS values increase and the RTS values decreased, the mortality rate increased. ROC analysis was performed to assess the correlation between the calculated risks and mortality. The calculated cut-off values and area under the curve (AUC) in ROC analysis was 14.5 (AUC=0.890) for ISS, 19 (AUC=0.896) for NISS, and 7.6 (AUC=0.943) for TRISS (*P*<0.001 for each). ROC curves of the three scoring systems are presented in Figure 1. The ideal cut-off value for RTS was 6.27 (AUC=0.915), and the reverse relation of the augmented score and diminished mortality is shown on a second ROC curve (Figure 2). Additionally, by performing a univariate logistic regression analysis, ISS (odds ratio [OR]: 0.261, 95% confidence interval [CI]: 1.125-1.498, *P*<0.001), NISS (OR: 0.180, 95% CI: 1.094-1.309, *P*<0.001), TRISS (OR: 0.004, 95% CI: 1.002-1.005, *P*<0.001), and RTS (OR: -0,001, 95% CI: 0.999-1.000, *P*<0.001) were significantly associated with mortality in all the penetrating cardiovascular injuries.

**Table 4:** Mean scores of all cardiovascular trauma patients, survivors, and nonsurvivors.

Parameters	All patients (mean±SD)	Survivor (mean±SD)	Nonsurvivor (mean±SD)	*P*-value
ISS	13.5±8.1	10.2±5.7	22.1±7.38	< 0.001
NISS	19.9±12.7	14.7±8.99	33.6±10.9	< 0.001
TRISS	24.7±39.9	6.9±21.7	72.3±38.8	< 0.001
RTS	5.7±3.0	7.09±1.8	2.15±2.7	< 0.001

The significance of scores for prediction of mortality is analyzed using Mann-Whitney U test ISS=Injury Severity Score; NISS=New Injury Severity Score; RTS=Revised Trauma Score; SD=standard deviation; TRISS=Trauma and Injury Severity Score

The mean risk scores of patients with and without cardiac injuries are shown separately in Table 5. The mortality rate of patients with penetrating cardiac injury was 53% (n=7/13). TRISS and RTS results correlated with mortality for cardiac injuries (Mann-Whitney U test, *P*=0.003 and *P*=0.01, respectively). ISS and NISS results were not correlated with mortality for cardiac injuries (Mann-Whitney U test, *P*>0.05) (Table 5). The cut-off values in cardiac injuries and in ROC analysis was 20.5 (AUC=0.786, *P*>0.05, sensitivity: 57%, specificity: 100%) for ISS, 22.5 (AUC=0.738, *P*>0.05, sensitivity: 85%, specificity: 50%) for NISS, 12.6 (AUC=0.929, *P*<0.05, sensitivity: 85%, specificity: 100%) for TRISS, and 5.61 (AUC=0.929, *P*<0.05, sensitivity: 85%, specificity: 100%) for RTS.

**Table 5:** Mean risk scores of patients with cardiac and vascular injuries.

	All patients (mean±SD)	Survivors (mean±SD)	Nonsurvivors (mean±SD)	*P*-value
Cardiac ınjury (n=13)				
ISS	20.38±7.06	16.000±0.000	24.143±8.009	0.035
NISS	29.07±9.46	24.67±8.16	32.86±9.35	0.132
TRISS	41.21±46.36	3.250±1.475	73.757±40.260	0.002
RTS	4.38±3.31	7.110±0.462	2.043±2.820	0.008
Vascular ınjury (n=64)				
ISS	12.1±7.67	9.580±5.679	21.143±7.145	< 0.001
NISS	18.04±12.53	13.580±8.401	34.000±12.006	< 0.001
TRISS	28.6±42.7	7.390±23.022	71.650±39.597	< 0.001
RTS	5.7±3.05	7.089±1.936	2.216±2.838	< 0.001

The validation of risk scores for mortality among cardiac (n=13) and vascular injuries (n=64) is made using the Mann-Whitney U test. ISS and NISS did not predict mortality for penetrating cardiac injuries (P>0.05, both) ISS=Injury Severity Score; NISS=New Injury Severity Score; RTS=Revised Trauma Score; SD=standard deviation; TRISS=Trauma and Injury Severity Score

Among 64 patients with vascular injury, mortality rate was 22% (n=14). ISS, NISS, TRISS, and RTS have shown a strong correlation with mortality in patients with vascular injuries (Mann-Whitney U test, *P*<0.001 for each) (Table 5). The calculated cut-off values and AUC in vascular injuries and in ROC analysis was 14.5 (AUC=0.896, *P*<0.001, sensitivity: 92%, specificity: 76%) for ISS, 17.5 (AUC=0.893, *P*<0.001, sensitivity: 78%, specificity: 64%) for NISS, 24.5 (AUC=0.946, *P*<0.001, sensitivity: 92%, specificity: 88%) for TRISS, and 6.74 (AUC=0.929, *P*<0.001, sensitivity: 85%, specificity: 100%) for RTS.

## DISCUSSION

Civilian cardiovascular trauma has always been a challenge for physicians, and it has been evaluated since the birth of cardiovascular surgery^[19]^. Victims of penetrating cardiovascular injuries mostly die on the scene or during transfer to a trauma facility. Overall survival has been reported between 11% and 73% for patients with cardiac injuries who arrive at a trauma center with signs of life^[20,21]^. Although overall mortality rates of 1.3%-10% for vascular trauma have been published in reports from high-level urban trauma centers, 13%-20% mortality rate range is reported in rural centers for vascular injuries, which is consistent with our results^[22,23]^. Our relatively high mortality rate (27% among all cases) is due to the high frequency of shotgun injuries and surgical management, including EDT, even if the patients had no vital signs and were resuscitated on arrival at the hospital. In our study, a 16% survival rate was found in EDT, similar to previously published survival rates^[24]^. The improvement of the ambulance system results in the arrival of more severely wounded patients at our hospital. Therefore, the overall inhospital mortality rate may increase. Of note, the support of a heart-lung machine was not available in the hospital during this study period. Although its use is reported to be occasional in trauma, it could improve the efficiency of the cardiovascular surgeon, especially in major cardiac injuries^[25]^.

To determine the efficacity of the allocated resources, as well as the training of health-care providers, the patient and treatment profile should be understood. With the use of an appropriate scoring system, the severity of trauma can be assessed, and a prediction of outcomes can be made on arrival at the hospital. In mixed trauma populations, the most commonly accepted threshold of ISS is 15, and it also indicates major trauma with substantially increased mortality, although higher cut-off values for mortality have been reported^[26,27]^. According to the analysis of nearly nine million trauma victims, 93% of urban and rural patients had ISS < 9, and less than 1% of the patients had ISS > 15^[28]^. The proportion of our patients with ISS > 15 (51%) was relatively high. This high proportion shows the more severe injuries to be dealt with when the cardiovascular system is involved. However, the mortality rate of a population with heterogeneous organ traumas and the results of trauma with the involvement of a special organ system might have different thresholds^[26,29,30]^.

When grouped as cardiovascular trauma, all of the studied scores were correlated with mortality using a Mann-Whitney U test (*P*<0.001 for each) and could be put in order as TRISS (AUC=0.943), RTS (AUC=0.915), NISS (AUC=0.896), and ISS (AUC=0.890) by decreasing force with regard to their AUCs (Table 4, Figures 1 and 2).

Subgroup analysis revealed that NISS had no correlation with mortality, while ISS had a lower significance (*P*=0.035) than TRISS (*P*=0.002) and RTS (*P*=0.008) by considering penetrating cardiac injuries (Table 5). A ROC analysis for cardiac injuries revealed that ISS and NISS had no significant effect on mortality prediction (*P*<0.05 for both). TRISS and RTS are more accurate regarding sensitivity, specificity, and AUC in ROC analysis involving penetrating cardiac injuries. This means that components of RTS (respiratory rate, besides blood pressure and Glasgow Coma Score) and TRISS (age) have a positive effect on outcome prediction. Hemodynamic status and physiological parameters at the time of admission in cardiac injuries seem to be better predictors of mortality, compared to anatomical region-based risk classifications. Topal et al.^[21]^ previously reported that TRISS was effective in predicting mortality in cardiac injuries in an urban center. The results of our study confirm the validity of TRISS and RTS with the high positive and negative predictive values in patients with penetrating cardiovascular trauma. Minor differences in favor of TRISS in terms of negative and positive predictive values may be related to the numerical expression of the results. RTS values are derived from a shorter range.

For vascular injuries, all risk scores were correlated with mortality using Mann-Whitney U test (*P*<0.001 for each) and ROC analysis *P*<0.001 for each) (Table 5).

## CONCLUSION

Penetrating cardiovascular trauma is one of the most significant life-threatening injuries. Geographic disparities, as well as increased firearm usage, may contribute to negative effects in lethal outcomes in rural areas. TRISS has shown the strongest correlation for predicting in-hospital mortality in victims of cardiovascular trauma. ISS and NISS did not correlate with the outcomes of cardiac injuries. TRISS and RTS can be used for assessment of the risk for mortality in victims of penetrating cardiovascular trauma.

**Table:** 

Authors' roles & responsibilities	
AAA	Substantial contributions to the conception or design of the work; or the acquisition, analysis, or interpretation of data for the work; final approval of the version to be published
ES	Substantial contributions to the conception or design of the work; or the acquisition, analysis, or interpretation of data for the work; final approval of the version to be published
FAB	Substantial contributions to the conception or design of the work; or the acquisition, analysis, or interpretation of data for the work; final approval of the version to be published
